# Supplementary material for: OpenMM 7: Rapid development of high performance algorithms for molecular dynamics
Source: PLoS Comput Biol. 2017 Jul 26;13(7):e1005659. doi: 10.1371/journal.pcbi.1005659 (PMC5549999; doi:10.1371/journal.pcbi.1005659)
Supplement: S1 Source Code — (ZIP) [file pcbi.1005659.s002.zip › OpenMM-7.0.1-Source/docs/api-c++/generated/OpenMM.LocalEnergyMinimizer.html]

LocalEnergyMinimizer — OpenMM @OPENMM\_MAJOR\_VERSION@.@OPENMM\_MINOR\_VERSION@ documentation


# LocalEnergyMinimizer¶

*class* `OpenMM::``LocalEnergyMinimizer`¶
:   Given a `Context`, this class searches for a new set of particle positions that represent a local minimum of the potential energy. The search is performed with the L-BFGS algorithm. Distance constraints are enforced during minimization by adding a harmonic restraining force to the potential function. The strength of the restraining force is steadily increased until the minimum energy configuration satisfies all constraints to within the tolerance specified by the `Context`‘s `Integrator`.

    void `minimize`(Context &*context*, double *tolerance* = 10, int *maxIterations* = 0)¶
    :   Search for a new set of particle positions that represent a local potential energy minimum. On exit, the `Context` will have been updated with the new positions.

        |  |  |
        | --- | --- |
        | Parameters: |  |

        - **context** – a `Context` specifying the `System` to minimize and the initial particle positions
        - **tolerance** – this specifies how precisely the energy minimum must be located. Minimization will be halted once the root-mean-square value of all force components reaches this tolerance. The default value is 10.
        - **maxIterations** – the maximum number of iterations to perform. If this is 0, minimation is continued until the results converge without regard to how many iterations it takes. The default value is 0.

High performance molecular simulation on GPUs


### Navigation

- Core Objects
- Forces
- Integrators
- Extras

©2015, Stanford University and the Authors.
|
Powered by Sphinx 1.3.1
& Alabaster 0.7.7
|
Page source
